# Liquid Chromatography-Tandem Mass Spectrometry (LC-MS/MS)-Based Proteomics of Drug-Metabolizing Enzymes and Transporters

**DOI:** 10.3390/molecules25112718

**Published:** 2020-06-11

**Authors:** Jiapeng Li, Hao-Jie Zhu

**Affiliations:** Department of Clinical Pharmacy, University of Michigan, Ann Arbor, MI 48109-1065, USA; ljiapeng@med.umich.edu

**Keywords:** proteomics, LC-MS/MS, drug-metabolizing enzymes, transporters

## Abstract

Liquid chromatography-tandem mass spectrometry (LC-MS/MS)-based proteomics is a powerful tool for identifying and quantifying proteins in biological samples, outperforming conventional antibody-based methods in many aspects. LC-MS/MS-based proteomics studies have revealed the protein abundances of many drug-metabolizing enzymes and transporters (DMETs) in tissues relevant to drug metabolism and disposition. Previous studies have consistently demonstrated marked interindividual variability in DMET protein expression, suggesting that varied DMET function is an important contributing factor for interindividual variability in pharmacokinetics (PK) and pharmacodynamics (PD) of medications. Moreover, differential DMET expression profiles were observed across different species and in vitro models. Therefore, caution must be exercised when extrapolating animal and in vitro DMET proteomics findings to humans. In recent years, DMET proteomics has been increasingly utilized for the development of physiologically based pharmacokinetic models, and DMET proteins have also been proposed as biomarkers for prediction of the PK and PD of the corresponding substrate drugs. In sum, despite the existence of many challenges in the analytical technology and data analysis methods of LC-MS/MS-based proteomics, DMET proteomics holds great potential to advance our understanding of PK behavior at the individual level and to optimize treatment regimens via the DMET protein biomarker-guided precision pharmacotherapy.

## 1. Introduction

Interindividual variability in drug disposition, i.e., absorption, distribution, metabolism, and excretion (ADME), is often associated with insufficient therapeutic effects and unexpected adverse drug events [1,2,3]. Drug-metabolizing enzymes and transporters (DMET) play major roles in drug disposition [4,5,6]; thus, interindividual variability in DMET functions may explain a large portion of the interindividual variability in drug disposition. Therefore, characterizing interindividual variability in DMET protein expression can provide insights into the variability of pharmacokinetics (PK) and further aid the development of precision pharmacotherapy.

Previous pharmacogenomics studies have identified numerous genetic variants associated with the expression and activity of DMET, and many genetic polymorphisms have been utilized in clinical practice to optimize pharmacotherapy [7]. However, a significant portion of DMET variability cannot be predicted by genetic variants, which is partially due to gene expression being regulated not only by genetic regulators, but also by various non-genetic factors (e.g., inducers) [8]. Furthermore, there is a growing body of evidence suggesting that mRNA expression correlates poorly with protein expression for many genes, including the majority of DMETs [9,10,11]. Thus, studying DMET protein expression could lead to better understanding of the effects of DMETs on PK and treatment outcomes, and the generated knowledge could be integrated into pharmacogenomics to further enhance precision pharmacotherapy.

Some conventional antibody-based assays, such as enzyme-linked immunosorbent assays (ELISAs) and Western blots, have been widely used for protein expression analysis. However, these methods depend on the availability and quality of specific antibodies, and it is frequently challenging to prepare highly selective antibodies for target proteins that share similar amino acid sequences [12]. Moreover, antibody-based analysis usually detects a limited number of proteins per assay, in most cases, one per analysis. In recent decades, liquid chromatography-tandem mass spectrometry (LC-MS/MS) has emerged as an important technique for protein identification and quantification. This technique has several advantages over conventional antibody-based assays. For instance, it is a high-throughput method with the ability to simultaneously quantify thousands of proteins [10,12,13]. Moreover, LC-MS/MS-based proteomics analysis can detect and quantify both native proteins and post-translational modifications (PTMs) [14], whereas antibody-based methods may require specific antibodies for each task.

In this review, we summarize recent progress in the LC-MS/MS-based proteomics of DMETs, with a focus on DMET protein expression profiles in human tissues relevant to PK, such as the liver, intestine, blood–brain barrier (BBB), and lung. Furthermore, we discuss the potential clinical applications of DMET proteomics, including the use of DMET protein expression data in the development of physiologically based pharmacokinetic (PBPK) models and the discovery of DMET protein biomarkers for precision pharmacotherapy.

## 2. LC-MS/MS-Based Proteomics Techniques for DMET Studies

An in-depth discussion of the principles of MS-based proteomics is beyond the scope of this review. We direct readers who have an interest in this topic to the recent publications [15,16]. Briefly, LC-MS/MS-based proteomics can be divided into two categories: “top-down” and “bottom-up”. The top-down strategy analyzes intact proteins without digestion. One of the advantages of “top-down” proteomics is its ability to detect and localize sequence variations, alternative splicing events, and PTMs, as this information is retained in intact proteins [17]. However, “top-down” proteomics has some limitations that hinder its application, which include low sensitivity, a limited number of bioinformatics tools, and inferior chromatographic performance of intact proteins compared to that of peptides [18]. Consequently, the use of “top-down” proteomics in ADME research is uncommon, and most DMET protein analyses were conducted using “bottom-up” methods. In “Bottom-up” proteomics, proteins are digested into peptides before being analyzed by LC-MS/MS. “Bottom-up” approaches can be further categorized into targeted and nontargeted (global) proteomics.

### 2.1. Targeted Proteomics

Targeted proteomics techniques detect the preselected surrogate peptides to quantify the proteins of interest. These techniques include selective reaction monitoring (SRM, also named multiple reaction monitoring, MRM) and parallel reaction monitoring (PRM, also named high-resolution multiple reaction monitoring, MRM-HR) modes. Targeted proteomics demonstrated higher sensitivity, specificity, and reproducibility than nontargeted proteomics due to its narrow isolation windows and specific peptides selected for monitoring. For SRM proteomics, both precursors and product ions are preselected, and ion fragmentation conditions need to be optimized, resulting in significant efforts in assay development [16,19]. As a comparison, PRM proteomics usually requires less effort in assay development because PRM uses a full scan of product ions from the preselected precursors [16,20]. PRM showed similar or even higher sensitivity than SRM for highly complex samples [21]. Additionally, PRM exhibited a higher selectivity than SRM because PRM proteomics is performed on a high resolution MS (e.g., time-of-flight and orbitrap MS) and SRM proteomics usually uses a low resolution MS (e.g., triple quadrupole instrument) [22]. It should be noted that the targeted proteomics techniques, including both SRM and PRM, have limited multiplexing capacity because each assay can only contain a finite number of target precursors [16,19]. Targeted proteomics techniques are considered as the gold standard for the quantification of selected sets of proteins [15]. For this reason, most DMET proteomics studies were performed using targeted proteomics techniques.

### 2.2. Non-Targeted Proteomics

#### 2.2.1. Data-Dependent Acquisition

Data-dependent acquisition (DDA), often termed as the “shotgun” approach, is characterized by selecting the most intensive precursors for fragmentation and analysis [15,23]. This technique can identify thousands of proteins in a complex biological sample but lacks reproducibility due to its inherent stochastic precursor selection [23]. DDA has been widely used in biomarker discovery since it is suitable for fast protein identification in complex protein mixtures [15]. The use of DDA in DMET research is relatively less frequent as DMET studies usually targeted a limited number of enzymes and transporters. A recent study employed a DDA method with an isobaric labeling technology to quantify human hepatic DMETs. The DDA results were highly correlated with the results obtained from a targeted proteomics study with the Pearson r value ranging from 0.74 to 1.00 for various DMETs [24]. In addition, this DDA technique provided broader proteome coverage than targeted proteomics methods [24].

#### 2.2.2. Data-Independent Acquisition

Data-independent acquisition (DIA) is a more recently developed non-target proteomics technology. In the DIA mode, precursors are separated into sequential small mass windows (5–25 Da), and all precursors in each mass window are then fragmented to acquire product ion spectra [25]. DIA is believed to own the combined strengths of SRM and DDA techniques regarding sensitivity, specificity [26,27], and proteome coverage [27]. Its ability to record all fragment ions of the precursors across mass windows enables it as a promising tool for biomarker discovery [28]. Overall, DIA is still a rapidly developing technology and has not been commonly used in DMET proteomics studies. Recently, several researchers compared the DIA technique with the conventional targeted proteomics methods for quantitative proteomics analysis. For instance, Nakamura et al. used a DIA technique to absolutely quantify 152 proteins, including many DMETs, in pooled human hepatic, intestinal, and renal microsomes and compared the results to those obtained from SRM and PRM analyses. The protein levels obtained by this DIA technique were highly correlated with the results from SRM and PRM analyses with a Pearson coefficient (r^2^) above 0.898, and the differences of the results between the DIA and targeted analyses were within 50% [29]. This study also demonstrated the superiority of DIA in the large-scale multiplex absolute quantification of DMETs to the targeted proteomics techniques [29]. Moreover, Jian et al. applied both DIA and PRM techniques to quantify drug-metabolizing enzymes (DMEs) in human livers and the human hepatic cell lines HepG2, Hep3B, and Huh7 and found a high correlation between the results obtained from DIA and PRM analyses, with r^2^ values ranging from 0.87 to 0.90 [30]. The performance comparison of SRM, PRM, DDA, and DIA is shown in Table 1 [15,16,26,27].

## 3. DMET Protein Expression Profiles Determined by LC-MS/MS-Based Proteomics

### 3.1. DMET Protein Expression in Human Tissues

#### 3.1.1. Human Hepatic DMET Protein Abundance

**Figure 1:**
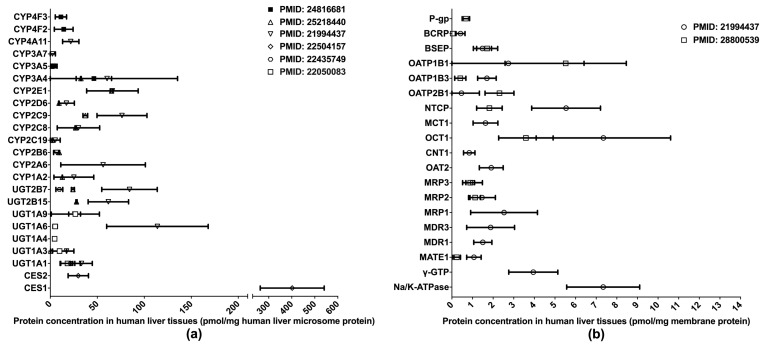
Absolute quantification of major clinically relevant human hepatic drug-metabolizing enzymes (**a**) and transporters (**b**). Protein expression levels are presented as means with standard deviations. CYP: cytochromes P450; UGT: uridine 5′-diphospho-glucuronosyltransferase; CES: carboxylesterase; P-gp, P-glycoprotein; BCRP, breast cancer resistance protein; BSEP: bile salt efflux pump; OATP: organic anion transporting polypeptide; NTCP: Na+-taurocholate co-transporting polypeptide; MCT: monocarboxylate transporter; OCT: organic cation transporter; CNT: concentrative nucleoside transporter; OAT: organic anion transporter; MRP: multidrug resistance-associated protein; MDR: multiple drug resistance; MATE: multidrug and toxin extrusion; γ-GTP: gamma glutamyl transpeptidase.

One of the primary applications of LC-MS/MS-based proteomics in the ADME field is the quantification of DMET proteins in various human tissues, especially those pertinent to drug metabolism and disposition, such as the liver, intestine, and blood–brain barrier (BBB). To date, the proteomics of hepatic DMETs has been extensively studied, and major clinically relevant DMETs have been quantified [10,13,31,32,33,34,35] (Figure 1). Among the quantified DMETs, carboxylesterase 1 (CES1) is the most abundant hepatic enzyme with a mean concentration of 400 pmol/mg total protein in human liver microsomes (HLM). Other abundant hepatic enzymes include UGT1A6, UGT2B7, CYP2C9, CYP2E1, and CYP3A4, with the mean values ranging from 50 to 150 pmol/mg in HLM. For transporters, OATP1B1, OCT1, NTCP, and MRP1 are among the most abundantly expressed in the liver. Notable interindividual variability was observed in the protein expressions of DMETs, suggesting a resource for the interindividual variability in the PK of their substrate drugs. Additionally, significant inter-study differences were evident for the quantification of many DMETs, including CYP2C9, UGT2B7, UGT2B15, NTCP, OATP1B3, OATP2B1, and MATE1. This discrepancy may be attributed to a variety of factors, such as differences in sample resources, sample preparation, and LC-MS/MS methods.

#### 3.1.2. Human Intestinal DMET Protein Abundance

**Figure 2:**
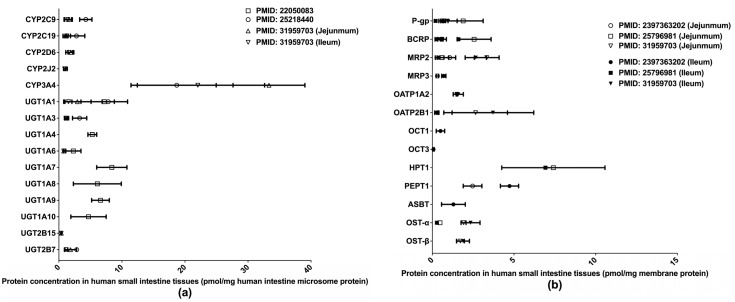
Absolute quantification of major clinically relevant human intestinal DMEs (**a**) and transporters (**b**). Protein expression levels are presented as means with standard deviations. CYP: cytochromes P450; UGT: uridine 5′-diphospho-glucuronosyltransferase; P-gp, P-glycoprotein; BCRP, breast cancer resistance protein; MRP: multidrug resistance-associated protein; OATP: organic anion transporting polypeptide; OCT: organic cation transporter; HPT: human peptide transporter; PEPT: Peptide transporter; ASBT: apical sodium–bile acid transporter; OST: organic solute transporter subunit.

Extant quantifications of human intestinal DMET proteins are summarized in Figure 2. Among those proteins, CYP3A4 and HPT1 are the most abundant enzyme and transporter, respectively. Two studies [32,33] determined the abundances of major clinically relevant DMEs in isolated human small intestine microsomes (HIMs) without regard to intestinal segments, whereas a more recent study [36] measured DME expression levels separately in the jejunum and ileum. Drug transporter protein expressions have likewise been measured separately in the jejunum and ileum [36,37,38]. A recent study [39] quantified multiple drug transporters in additional intestinal segments, including the duodenum, jejunum, ileum, and colon, and identified a segment-dependent expression pattern for the transporters [39]. The differences in drug transporter expression across intestinal segments may contribute to varied absorption rates for oral drugs at different intestinal segments and lead to the multiple-peaking phenomenon observed in the plasma drug concentration-time profiles of some medications [40]. Proteomics data describing the intestinal transporter distribution would be of great help in understanding drug oral absorption features and in optimizing the designs of drug delivery systems and formulations.

#### 3.1.3. DMET Proteins Expressed at the Human BBB

**Figure 3:**
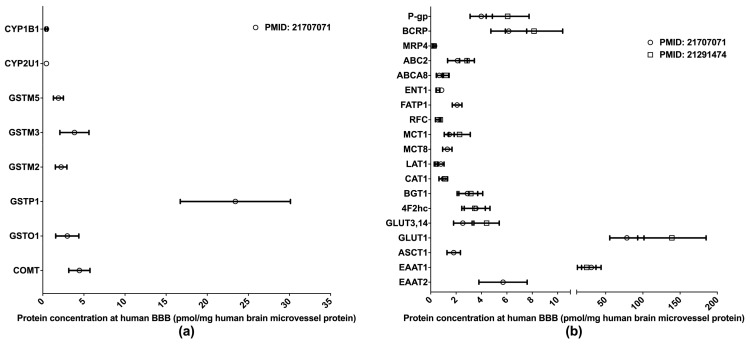
Absolute quantification of major clinically relevant drug-metabolizing enzymes (**a**) and transporters (**b**) at human blood–brain barrier (BBB). Protein expressions are presented as means with standard deviations. CYP, cytochrome P450; GSTM: glutathione S-transferase-Mu; GSTO: glutathione S-transferase omega; GSTP: glutathione S-transferase P; COMT: catechol O-methyltransferase; P-gp: P-glycoprotein; BCRP: breast cancer resistance protein; MRP: multidrug resistance-associated protein; ABC2: ATP-binding cassette transporter 2; ABCA8: ATP-binding cassette sub-family A member 8; ENT1: equilibrative nucleoside transporter 1; FATP1: Fatty acid transport protein 1; RFC: reduced folate carrier; MCT: monocarboxylate transporter; LAT: L-type amino acid transporter; CAT: cationic amino acid transporter; BGT: betaine-GABA transporter; 4F2hc: 4F2 heavy chain; GLUT: glucose transporter; ASCT: alanine–serine–cysteine transporter; EAAT: excitatory amino acid transporter.

The BBB is formed of the endothelial cells of brain microvessels and characterized by the presence of intercellular tight junctions. The BBB was previously viewed as an anatomical barrier between the blood and the brain [41] and is now also considered a pharmacological barrier because a range of DMETs are expressed at the BBB. These DMETs are involved in drug metabolisms and the uptake and efflux of a variety of drugs across the BBB, hence affecting drug distribution in the brain [42,43]. Brain drug distribution is associated with the efficacy and side effects of drugs for the treatment of many diseases, such as brain tumors, HIV-1 infection, and psychiatric disorders [44]. Therefore, obtaining DMET protein expression profiles at the BBB will provide more insights into the role of the BBB in regulating the pharmacological effects of central nervous system medications; and the information will also aid in drug development and the optimization of pharmacotherapy. Relative to human hepatic and intestinal DMET proteomics, the proteomics of human BBB DMETs was understudied. The quantifications of clinically relevant DMETs at the human BBB are illustrated in Figure 3. One study determined the abundance of several DMEs [45], and two reported the levels of major transporters [45,46]. Among the reported DMETs, the phase II enzyme GSTP1 exhibited a considerably higher level of expression. GLUT1 and EAAT1 were found to be the two most abundant uptake transporters, whereas BCRP and P-gp were among the most abundant efflux transporters.

#### 3.1.4. DMET Proteins Expressed in Human Lungs

**Figure 4:**
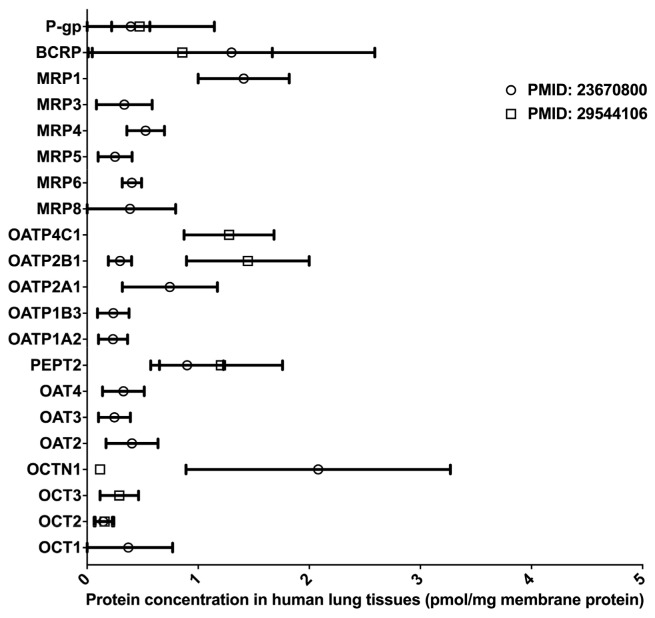
Absolute quantification of major clinically relevant transporter proteins in human lungs. Protein expressions are presented as means with standard deviations. BCRP, breast cancer resistance protein; P-glycoprotein; MRP, multidrug resistance-associated protein; OATP, organic anion transporting polypeptide; PEPT, peptide transporter; OAT, organic anion transporter; OCTN, organic cation/carnitine transporter; OCT, organic cation transporter.

Inhaled drugs are commonly used for the treatment of pulmonary diseases, such as asthma, chronic obstructive pulmonary disease, and lung cancer. Inhalation administration features rapid absorption, quick pharmacological action onset in the lung, and improved bioavailability through the avoidance of the fast-pass effect caused by metabolism in the liver and intestine [47]. Many DMETs, including CYPs, UGTs, CESs, and uptake and efflux drug transporters, have been detected in the lung tissues, and these DMETs are essential in governing the absorption and distribution of inhaled medications [48,49,50,51,52]. However, to date, LC-MS/MS-based proteomics studies for the determination of absolute DME protein levels in the lung have been lacking. Two proteomics studies quantified the protein expressions of multiple drug transporters in the lung [50,51] (data summarized in Figure 4). Notably, the protein expression profiles of drug transporters in the lung were distinct from that in the liver and intestine [51]. In the lung, BCRP, P-gp, and MRP1 were the most abundant efflux transporters, whereas OATP 2A1, 2B1, and 4C1, and PEPT represented the most abundant uptake transporters. Overall, significant interindividual variability in transporter expression was observed, suggesting that lung transporters are a potential contributor to interindividual variability in the PK and pharmacological effects of inhaled drugs [50]. In addition, there was a significant inter-study difference in the abundance of OCTN1 and OATP2B1, probably due to differing sample resources and different platforms used in the proteomics analyses. It should be noted that both studies had a relatively small sample size (*n* < 7). Given the scarcity of quantitative proteomics data for human lung DMETs, more LC-MS/MS-based proteomics studies are warranted, and the results will help reveal the impact of DMETs on the metabolism and disposition of inhaled medication in the lung.

### 3.2. Differential DMET Protein Expression Across Different Species and Cell Lines

Investigators have conducted comparative proteomics analyses of DMET proteins in samples across human subjects, animal models, and various cell lines; these analyses revealed significant inter-species and inter-model differences (Table 2). For instance, Shi et al. used both global and targeted proteomics methods to compare the abundance of multiple clinically relevant DMEs in human livers with that in the commonly used human hepatic cell lines HepG2, Hep3B, and Huh7 [30]. A majority of targeted DMEs were not detected in the cell lines, and for those that were, the protein expression levels were significantly lower than in human livers [30]. Yasuo et al. quantified major drug transporters in human and mouse BBB using a selective-reaction monitoring proteomics approach. The study showed that BCRP and P-gp protein levels were 1.85-fold higher and 2.33-fold lower, respectively, in human BBB than in mouse BBB. Additionally, a majority of the organic anion transporters (OATs), organic anion transporting polypeptides (OATPs), and multidrug resistance-associated proteins (MRPs) found in mouse BBB were below the limit of quantification in human BBB [46]. Another proteomics study demonstrated considerable differences in the abundances of several transporters between human brain microvessels and the hCMEC/D3 cell line, an established human BBB in vitro model [53]. These differential DMET protein expression patterns between species and between in vivo and in vitro models suggest that caution needs to be exercised when applying in vitro and animal models to PK research; the differing DMET expression levels in these models have to be taken into account during the extrapolation of in vitro and animal proteomics data to humans. Further investigation is required to identify in vitro and animal models that more closely resemble the protein expression patterns of DMETs in human bodies.

### 3.3. Proteomics of DMET Isoforms

Alternative splicing occurs during the transcriptional process and produces multiple transcripts, which consequently give rise to multiple protein products with distinct structures and functions [57]. Approximately 95% of human multi-exon genes contain alternative splicing phenomena, and this is considered to be an essential source of the functional diversity of genes [58]. By extension, alternative splicing may result in multiple isoforms of a DMET, which could contribute to interindividual variability in DMET functions and the ensuing PK. Alternative splicing variants have been documented for DMETs, such as CYPs [59], UGTs [60], and ABC and SLC transporters [61]; however, most previous alternative splicing studies were conducted at the mRNA level through the characterization of transcriptome profiles [59,60,61]. Since the correlations between mRNA expression and protein expression are poor for many DMETs [9,10,11], the influence of alternative splicing variants on DMET function may need to be evaluated at the protein level using appropriate proteomics assays. For example, human CES1 protein has four isoforms due to alternative splicing. A recent study applied an LC-MS/MS proteomics technique to quantify the abundances of all four CES1 isoforms in transfected cell lines and human liver samples [62]. The results showed that isoforms 1 and 2 constituted 74–90% of total CES1 expression and were highly correlated with total CES1 activity in human livers, whereas isoforms 3 and 4 were minor isoforms and had limited contributions to total CES1 expression and activity. Therefore, interindividual variability in the relative abundance of CES1 isoforms in the liver may contribute to variability in the PK of many CES1 substrates [62]. We expect that similar isoform-specific proteomics approaches will be utilized for the study of the isoforms of other DMETs in the future.

### 3.4. Post-Translational Modifications and Protein–Protein Interactions

Many proteins undergo modifications and interactions following their biosynthesis, giving rise to a new level of diversity of proteins and protein isoforms. LC-MS/MS-based proteomics techniques can identify PTMs and protein–protein interactions (PPIs), allowing for a better understanding of protein functions in physiological processes and diseases [63,64]. To date, however, most LC-MS/MS-based proteomics studies for PTMs [63,65,66] and PPIs [64,67] focused on proteins that are relevant to pathogenesis, disease diagnosis, and drug targets, while the studies on the PTMs and PPIs of DMETs were relatively limited. A recent DDA study investigated DME sulfenylation in human liver and kidney using an isotope-coded dimedone/iododimedone labeling strategy [68]. Sulfenylations were identified in human liver and kidney microsomes for many DMEs, including CYPs, UGTs, CESs, monoamine oxidases, flavin-containing monoxygenases (FMOs), and aldehyde dehydrogenases. Further catalytic activity and spectral analyses of CYP1A2, 2C8, 2D6, and 3A4 confirmed the sulfenylations and revealed two categories of redox sensitivity in these enzymes: heme-thiolate-sensitive and thiol-insensitive, suggesting a PTM regulatory mechanism for CYPs and other DMEs [68]. For PPIs, a recent DDA study established an affinity purification method to characterize the endogenous protein interactome of UGT1A enzymes in human liver, intestine, and kidney [69]. Several transferases, transporters, and dehydrogenases that are important for the metabolism of small lipophilic molecules and drugs were found to have a significant interaction with UGT1A enzymes. Several enzymes involved in the fatty acid β-oxidation, glycolysis, and glycogenolysis pathways were also identified as interactors to UGT1A enzymes, suggesting an impact of PPI on the glucuronidation and bioenergetic metabolism [69]. Overall, LC-MS/MS-based proteomics techniques, especially untargeted methods, have played an important role in the identification of PTMs and PPIs with high efficiency and confidence. Coupled with other techniques, such as protein purification and activity and spectral evaluations, LC-MS/MS-based proteomics is expected to identify more PTMs and PPIs involved in the regulation of DMET functions.

### 3.5. Enzyme Induction

The evaluation of DMET induction is indispensable to drug–drug interaction investigations, which are essential for drug development. Previous DMET induction studies were often conducted at the mRNA expression level. However, mRNA expressions were correlated poorly with protein expressions for many DMEs [10,70]. Several recent studies applied targeted proteomics techniques to determine the changes in DMET protein expressions in response to different inducers, including antibiotics [71], rifampicin [72], corticosteroids [73], and cholic acid [74]. Most of the induction studies were performed in mice, rats, and other animal models, and DMET protein induction in humans remains largely unexplored. Of note, a recent study developed a method combining a targeted proteomics method and immunoprecipitation to quantify DMEs in human hepatocytes [70]. Findings from this study revealed the inducible effects of omeprazole, phenobarbital, and rifampicin on the protein expression of CYP1A2, 2B6, 3A4, and 2C8. Furthermore, CYP protein levels could better reflect the induction of CYP activities than mRNA levels [70], suggesting that, relative to mRNA quantification, protein quantification may better predict the in vivo functional changes of induced DMETs.

## 4. Potential Clinical Applications of DMET Proteomics

### 4.1. Integration of DMET Proteomics with PBPK Modeling

One of the practical applications of DMET proteomics is to identify the contributors affecting DMET protein expression and then apply that information to the development and optimization of PBPK models. PBPK modeling has been widely used in drug development and precision pharmacotherapy to predict PK by taking into account various factors involved in the process of drug metabolism and disposition. Therefore, integrating proteomics data into PBPK modeling offers a promising technique for improving PK prediction in patients with varying physiological and pathological conditions [75,76]. Many genetic and non-genetic factors capable of regulating DMET protein expression have been identified through LC-MS/MS-based proteomics studies (Table 3), including genetic variants, age, gender, and disease condition. Recently, researchers successfully developed PBPK models using quantitative DMET proteomics data. Mikael and colleagues incorporated the age-dependent protein expression data of CES1 and CES2 into a pediatric PBPK model to describe the PK of the anti-influenza prodrug oseltamivir. The predicted PK parameters, including area under the curve (AUC), maximal plasma drug concentration (C_max_), and time for C_max_ (T_max_), were within 2.1-fold of the clinically observed values [77]. Bhagwat et al. identified significantly lower protein expression levels of OATP1B1 in SLCO1B1 *14 carriers relative to subjects with the reference *1a/*1a genotype and used the Simcyp software to embed the proteomics data into PBPK models for rosuvastatin and repaglinide [78]. The models predicted an up to 40% lower AUC of rosuvastatin and repaglinide in patients with the SLCO1B1 *14/*14 genotype compared to those carrying wild type SLCO1B1, which was in agreement with the observed clinical data [78]. In another study, Bhagwat and colleagues demonstrated significantly lower expression of UGT2B7 protein in cirrhotic livers when compared to healthy controls. Subsequently integrating the proteomics data into a pediatric liver cirrhosis PBPK model improved the prediction of zidovudine and morphine PK in this patient population [79].

It should be noted that DMET expression could be affected by the interactions of multiple regulating factors, making it challenging to incorporate these contributors into a PBPK model. For example, in a study evaluating the effects of age and genotype on protein expression of hepatic UGTs, a significant association between age and UGT1A1 protein expression was found only in HLM samples with the reference UGT1A1 *1/*1 genotype, not in samples carrying UGT1A1 polymorphisms; this suggested an interplay between ontogeny and genotype that affects UGT1A1 expression. Similarly, in another study, researchers did not find an age-dependent OATP1B1 expression pattern in HLM samples with mixed *SLCO1B1* genotypes, but did observe an age effect when samples carrying *SLCO1B1* genetic variants were excluded from the data analysis [80]. Moreover, samples with the SLCO1B1*14/*1A genotype showed a significantly higher OATP1B1 protein abundance than did SLCO1B1*15/*1A carriers within the same age group (>1 year). These studies suggest that genotypes could confound the developmental expression of DMETs, and thus, genotyping information could be pertinent to the development of PBPK models involving certain DMETs [80].

### 4.2. DMET Protein Biomarkers for Precision Pharmacotherapy

Knowing DMET expression levels in organs involved in drug metabolism and disposition (e.g., the liver) would be invaluable for the prediction of PK and PD and the optimization of pharmacotherapy. However, tissue biopsy in the clinic is usually impractical due to its invasive nature. In contrast, human blood is among the most accessible clinical samples and contains a wealth of information about the health state of an individual [89]. Previous proteomics studies have identified many blood protein biomarkers for the diagnosis and treatment of cardiovascular diseases [90] and cancers [91], but efforts to establish ADME protein biomarkers in blood have been limited. Recently, DMET proteins in exosomes isolated from human plasma were proposed as a potential biomarker for PK prediction and pharmacotherapy optimization [92]. Human plasma exosomes are membrane-enclosed particles secreted from different organs (e.g., the liver and kidney). Since plasma exosomes carry nucleic acids, proteins, and lipids from the originating organs [93], the exosomes could serve as surrogates for investigating the functional status of those organs [92,93]. Recently, researchers used an LC-MS/MS-based proteomics method to detect the peptides of many CYPs (CYP 1A2, 2B6, 2C8, 2C9, 2C19, 2D6, 2E1, 2 J2, 3A4, and 3A5) and UGTs (UGT 1A1, 1A3, 1A4, 1A6, 1A9, 2B4, 2B7, 2B10, and 2B15) in human plasma exosomes [94]. They also observed an inductive effect of rifampicin on CYP3A4 mRNA expression, protein expression, and activity in both exosomes and liver samples. Interestingly, the mRNA and protein expressions and catalytic activity of CYP3A4 in the exosomes were highly correlated to the oral clearance of the CYP3A substrate midazolam, suggesting that CYP3A4 in plasma exosomes could serve as an in vivo biomarker for the PK of its substrate drugs [94]. It should be noted that the exosomal CYP3A4 protein levels were determined by an ELISA assay instead of an LC-MS/MS-based proteomics method in the study. While the concept of using plasma exosomes for the discovery of DMET biomarkers is intriguing, the lack of a standardized method for exosome isolation and a highly sensitive and reproducible proteomics method for exosomal DMET quantification remains as a major obstacle in the development of exosomal DMET biomarkers for pharmacotherapy optimization [92].

### 4.3. Challenges for DMET Proteomics Assay Development and Applications

Quantifying low abundance DMET proteins in complex biological samples, such as human plasma, has posed a challenge for proteomics assay development [95]. Several protein enrichment techniques have been used to improve the sensitivity of proteomics analysis of low abundance proteins, including the depletion of high-abundance proteins, immunoprecipitation, and multidimensional LC analysis [96]. However, these protein enrichment techniques come with several limitations. For instance, the depletion and immunoprecipitation techniques usually require antibodies, which are associated with the high cost and poor reproducibility [96,97]. Moreover, the multidimensional LC strategy is labor-intensive and demands extensive instrument time, thus limiting its broad applications.

Despite the tremendous potential of DMET proteomics in broad clinical applications, numerous challenges need to be overcome before the technology can become a reliable tool in clinical research and practice. (1) To date, most DMET proteomics studies have been conducted in in vitro settings. While in vitro to in vivo extrapolation may facilitate the validation and clinical adoption of DMET protein biomarkers, the utility of the biomarkers discovered in in vitro investigations needs to be validated through clinical trials. (2) Building protein biomarkers-guided PBPK models often requires high-quality proteomics data from a large number of patient samples [75]. However, accessing high-quality human tissues could be challenging, especially from special patient populations (e.g., pediatric patients). In fact, most pediatric DMETs proteomics studies used autopsy tissues, which imposes concerns about sample quality as some proteins may have degraded prior to analysis [80]. (3) Proteomics studies usually generates vast amounts of data and requires highly sophisticated data management and analysis [98]. Additionally, the lack of a standardized protocol for sample preparation and analytical method development has led to inconsistent results across different laboratories [8,98]. Therefore, the standardization of LC-MS/MS techniques and data analysis in the field of DMET proteomics is urgently needed. (4) The high instrumentation cost and the steep learning curve of LC-MS/MS-based proteomics represent additional barriers hindering the wide adoption of this technology in clinical applications [99]. Nevertheless, with rapid advances in LC-MS/MS-based proteomics, we expect that the abovementioned issues will be addressed, and DMET proteomics will become widely utilized as an important tool for predicting PK and optimizing drug treatment.

## 5. Conclusions

LC-MS/MS-based proteomics has been used extensively for the quantification of clinically relevant DMETs in human and animal tissues and in in vitro models. DMET proteomics could play a role complementary to DMET pharmacogenomics and enable better understanding of interindividual variability in DMET function. Integrating DMET proteomics data into PBPK models is likely to improve the prediction of PK profiles. Moreover, DMET proteomics of plasma exosomes has shown the potential to predict in vivo DMET functions and the PK of the corresponding substrates. We envision that, with the ongoing rapid advances in LC-MS/MS-based proteomics, DMET proteomics will be increasingly used as a versatile tool to study interindividual variability in PK and identify DMET protein biomarkers for precision pharmacotherapy.

## Figures and Tables

**Table 1:** Comparisons of the performance of selective reaction monitoring (SRM), parallel reaction monitoring (PRM), data-dependent acquisition (DDA), and data-independent acquisition (DIA)^a^.

Techniques	Performance					
	Sensitivity	Specificity	Reproducibility	Multiplexing	Assay development	Prevalence in DMET study^b^
SRM	+ + +	+ + +	+ + + +	+	+	+ + + +
PRM	+ + + +	+ + + +	+ + + +	+	+ +	+ +
DDA	+	+	+	+ + + +	+ + + +	+
DIA	+ +	+ +	+ +	+ + + +	+ + +	+

^a^The number of “+” indicates the performance of a specific technique with “+ + + +” denoted to the best performance and “+” for the lowest performance. ^b^ The prevalence of each technique used in drug-metabolizing enzymes and transporters (DMET) proteomics research was estimated from the literature collected by the writing of this paper (May, 2020).

**Table 2:** Differences in DMET abundance across species and cell lines.

DMET	Tissue	Main Results	Year of Publication	Reference
CYPs, UGTs, CESs	Liver	Significant lower expression levels of major clinically-relevant DMEs were observed in the microsomes of HepG2, Hep3B, and Huh7 cell lines relative to human liver samples.	2018	[30]
BCRP, BSEP	Liver	The abundance of BCRP/Bcrp and BSEP/Bsep in the livers and isolated hepatocytes from different species (dog, rat, monkey, and human) were characterized.	2009	[54]
Transporters	Liver, Kidney	The differences in the abundance of four efflux transporters, including MDR1/P-gp, BCRP/Bcrp, MRP2/Mrp2, and MRP3/Mrp3, in the liver and kidney between different species (dog, rat, monkey, and human) were characterized.	2016	[55]
Transporters	BBB	Significant differences in protein expression levels of major drug transporters were identified between human and rodent BBB.	2011	[46]
Transporters	BBB	The protein expression levels of major drug transporters differed significantly among human cerebral microvascular endothelial cell line (hCMEC/D3), human brain microvessels, and human umbilical vein endothelial cells (HUVECs).	2012	[53]
Transporters	blood-retinal barrier	Transporters were differentially expressed between ARPE19 and hfRPE cells, the commonly used cellular models for human RPE.	2017	[56]

DME: drug-metabolizing enzyme; BBB: blood-brain barrier; CYP, cytochrome P450; UGP, uridine-diphosphate glucuronosyl transferase; CES, carboxylesterases; BCRP, breast cancer resistance protein; BSEP, bile salt efflux pump.

**Table 3:** Regulating factors contributing to interindividual variability in DMET protein expression.

DMET	Tissue	Regulators	Main Findings	Year of Publication	Reference
CYPs, UGTs	Liver	CYP3A5 genotype	Significant correlations in protein expression were found for UGT1A6/UGT1A9, UGY2B4/UGT2B15, and CYP1A2/UGT2B4; the CYP3A5 protein expression levels in subjects with the *1/*3 genotype were higher than that with *3/*3.	2014	[81]
CYPs, UGTs, and transporters	Liver	CYP3A5 genotype	Gender had negligible effect on the target DME expression in the liver. The expressions of all DMEs showed an overall decrease trend with age. The protein abundance of CYP3A5 in the livers with the *1/*3 genotype was 16-fold higher than that with the *3/*3 genotype. DMETs expression levels showed an overall trend of decrease with increasing BMI.	2019	[82]
OATPs, P-gp	Liver	SLCO1B1 genotype	The protein levels of OATP1B1 in the livers carrying the SLCO1B1 *14/*14 and *14/*1a genotypes were significantly higher than that with *1a/*1a. SLCO1B3 SNPs had an insignificant impact on the protein expression of OATP1B3.	2013	[78]
Transporters	Liver	SLCO1B1 genotype and Age	The protein expression levels of OCT1, OATP1B3, P-gp, and MRP3 in HLM increased with age. Gender had a negligible impact on the protein abundance of the hepatic transporters. In liver samples with ages >1 year, SLCO1B1*14/*1A was associated with 2.5-fold higher OATP1B1 protein expression relative to SLCO1B1*15/*1A carriers.	2016	[80]
UGTs	Liver	Age and genotype	The protein expressions of UGT1A1, UGT1A4, UGT1A6, UGT1A9, UGT2B7, and UGT2B15 in HLM were age-dependent, increasing from neonatal to adulthood. UGT1A1 protein expression was affected by multiple SNPs and was regulated by the ontogeny-genotype interplay phenomenon. rs1902023 (*2) carriers showed a decreased enzymatic activity but a comparable protein expression level of UGT2B15.	2019	[83]
CES1 and CES2	Liver	Age	The protein expression levels of CES1 and CES2 in adults were nearly 5-fold and 3-fold higher, respectively, than those in neonates.	2017	[77]
Transporters	Liver	Age	Fetal livers exhibited lower protein levels of BSEP, MDR1, MRP1, MRP2, MRP3, and OCT1, but higher protein expression levels of GLUT1 and OATP1B1 than adult samples. Age showed an insignificant impact on the protein abundance of ATP1A1, BCRP, MCT1, OATP1B3, and OATP2B1.	2018	[84]
OAT2 and OAT7	Liver	Age and gender	Age and sex did not affect the protein levels of OAT2 and OAT7 in the liver. A positive correlation in protein expression was observed between these two transporters.	2018	[85]
Transporters	Lung	Gender	MRP1 expression levels in the bronchial region showed high interindividual variability. The protein expression levels of MRP3, MRP5, MRP8, OCT1, and OCTN1 in females were significantly higher than in males.	2013	[50]
CYPs and UGTs	Liver	HBV-Positive Human Hepatocellular Carcinoma	The protein abundance of eight CYPs (CYP1A2, 2A6, 2B6, 2C8, 2C9, 2C19, 2D, 2E1, and 3A4) and three UGTs (UGT 1A1, 1A4, and 2B7) were significantly lower in tumor microsomes.	2015	[86]
SULT	Liver	Hepatocellular carcinoma	Hepatocellular carcinoma exhibited a significantly reduced level of protein expression of SULTs.	2017	[87]
CYPs, ADHs, UGTs, CESs	Liver	Alcoholic or hepatitis C cirrhotic	Cirrhosis did not affect hepatic CES2 protein expression. The protein abundance of most other DMEs were significantly lower in cirrhotic livers compared to healthy controls.	2018	[79]
CYPs, UGTs, transporters	Jejunum	BMI, smoke	A positive correlation between the expressions of CYP1A2 and GLUT4 and BMI was identified. Higher protein expression levels of UGT1A1 and UGT1A3 were observed in smokers.	2018	[88]

CYP, cytochrome P450; UGP, uridine-diphosphate glucuronosyl transferase; CES, carboxylesterases; P-gp, P-glycoprotein; MRP, multidrug resistance protein; BCRP, breast cancer resistance protein; OCT: organic cation transporter; OATP, organic anion transporting polypeptide; MCT, monocarboxylate transporter; PEPT, peptide transporter; GLUT1, glucose transporter 1; NTCP, Na+-taurocholate co-transporting polypeptide; BSEP, bile salt efflux pump; SULT, sulfotransferase; ADH, alcohol dehydrogenase; AOX, aldehyde oxidase; HLM, human liver microsomes; GST, glutathione S-transferases; COMT catechol O-methyltransferase; MATE, multidrug and toxin extrusion.
